# Fine Physical Bin Mapping of the Powdery Mildew Resistance Gene *Pm21* Based on Chromosomal Structural Variations in Wheat

**DOI:** 10.3390/ijms19020643

**Published:** 2018-02-24

**Authors:** Shanying Zhu, Yaoyong Ji, Jian Ji, Tongde Bie, Anli Gao, Huagang He

**Affiliations:** 1 School of Food and Biological Engineering, Jiangsu University, Zhenjiang 212013, China; zhushanying@mail.ujs.edu.cn (S.Z.); 2211618014@stmail.ujs.edu.cn (Y.J.); jijian221118005@163.com (J.J.); 2 School of Environment, Jiangsu University, Zhenjiang 212013, China; 3 Yangzhou Academy of Agricultural Sciences, Yangzhou 225007, China; btd@wheat.org.cn; 4 School of life Sciences, Henan University, Kaifeng 475004, China

**Keywords:** *Pm21*, powdery mildew resistance, physical mapping, comparative mapping

## Abstract

*Pm21*, derived from wheat wild relative *Dasypyrum villosum*, is one of the most effective powdery mildew resistance genes and has been widely applied in wheat breeding in China. Mapping and cloning *Pm21* are of importance for understanding its resistance mechanism. In the present study, physical mapping was performed using different genetic stocks involving in structural variations of chromosome 6VS carrying *Pm21*. The data showed that 6VS could be divided into eight distinguishable chromosomal bins, and *Pm21* was mapped to the bin FLb4–b5/b6 closely flanked by the markers 6VS-08.6 and 6VS-10.2. Comparative genomic mapping indicated that the orthologous regions of FLb4–b5/b6 carrying *Pm21* were narrowed to a 117.7 kb genomic region harboring 19 genes in *Brachypodium* and a 37.7 kb region harboring 5 genes in rice, respectively. The result was consistent with that given by recent genetic mapping in diploid *D. villosum*. In conclusion, this study demonstrated that physical mapping based on chromosomal structural variations is an efficient method for locating alien genes in wheat background.

## 1. Introduction

Common wheat (*Triticum aestivum* L.) is one of the most important cereal crops. Wheat production is seriously threatened by powdery mildew caused by *Blumeria graminis* f. sp. *tritici* (*Bgt*). The powdery mildew resistance gene *Pm21*, originating from *Dasypyrum villosum* Candargy (2*n* = 14, VV), confers highly effective resistance to all known isolates of *Bgt* [1,2]. As the donor of *Pm21*, wheat-*D. villosum* translocation line T6AL.6VS has been widely applied in wheat breeding. More than 20 varieties with this translocated chromosome have been planted on an accumulated area of more than four million hectares in China [3]. However, up to now, little is known about the nature of *Pm21* and its resistance mechanism. Therefore, it is essential to map and clone *Pm21*.

Previously, *Pm21* had been mapped to the chromosomal bin FL0.45–0.58 of 6VS using two deletion lines del.6VS-1 (FL0.58) and del.6VS-2 (FL0.45) [2,4]. In the recent study, 6VS FL0.45–0.58 was verified to be closely flanked by the markers 6VS-03 and 6VS-23 via physical mapping and comparative mapping, which narrowed the orthologous regions of FL0.45–0.58 to 1.06 Mb in *Brachypodium* and 1.38 Mb in rice, and involved 219 and 328 putative genes, respectively [5]. Nevertheless, the bin FL0.45–0.58 is still a large chromosomal segment and insufficient for fine mapping *Pm21*. Thus, it remains difficult to physically map *Pm21* further. 

In 2013, two small alien translocation lines NAU418 and NAU419 resistant to powdery mildew were developed by irradiation, both of which carry relatively short segments conveying *Pm21* [6]. Moreover, a susceptible deletion line Y18-S6 was recently obtained from ethyl methanesulfonate (EMS)-induced Yangmai 18, a wheat variety carrying translocated chromosome T6AL.6VS [7]. All of them are potential genetic materials for more fine physical mapping of *Pm21*. In this study, we attempted to physically map *Pm21* based on the above genetic stocks. The data obtained here will give a new insight into the chromosome localization of *Pm21*.

## 2. Results

### 2.1. Physical Mapping of Pm21

In the present study, a total of 45 gene-derived 6VS markers were used to map *Pm21*. Among them, 42 markers were reported previously, including 6VS-01–6VS-30 [5], 6VS_-381_ [8], MBH1 [3], Xcinau188, Xcfe164 [9], Xcau127 [10], CINAU15m, 6VS-08.4, 6VS-08.8, 6VS-10.2, 6VS-10.4, 6VS-10.6, and 6VS-10.8 [7]. The other three markers—6VS-08.6, 6VS-12.5, and 6VS-13.5—were newly developed in this study. 

**Figure 1:**
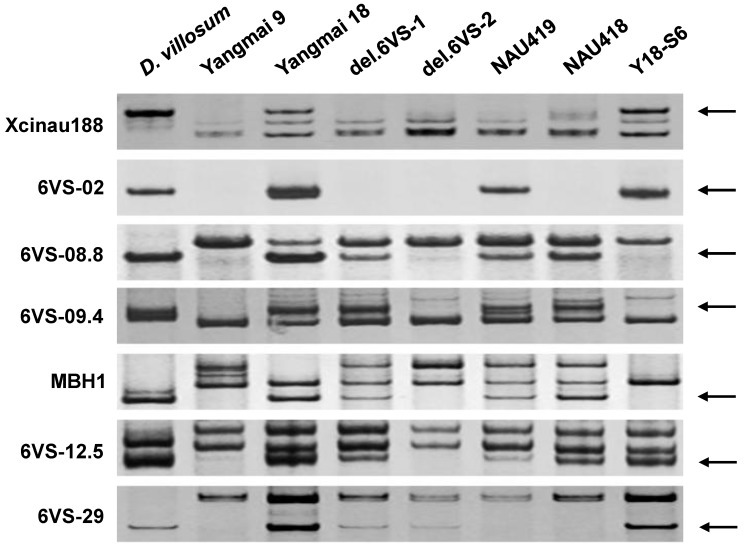
PCR amplification patterns of seven representative markers. The DNA templates used are labeled on the gel, including *D. villosum*, Yangmai 9, Yangmai 18, del.6VS-1, del.6VS-2, NAU419, NAU418, and Y18-S6. The polymorphic DNA bands are pointed by arrows.

**Figure 2:**
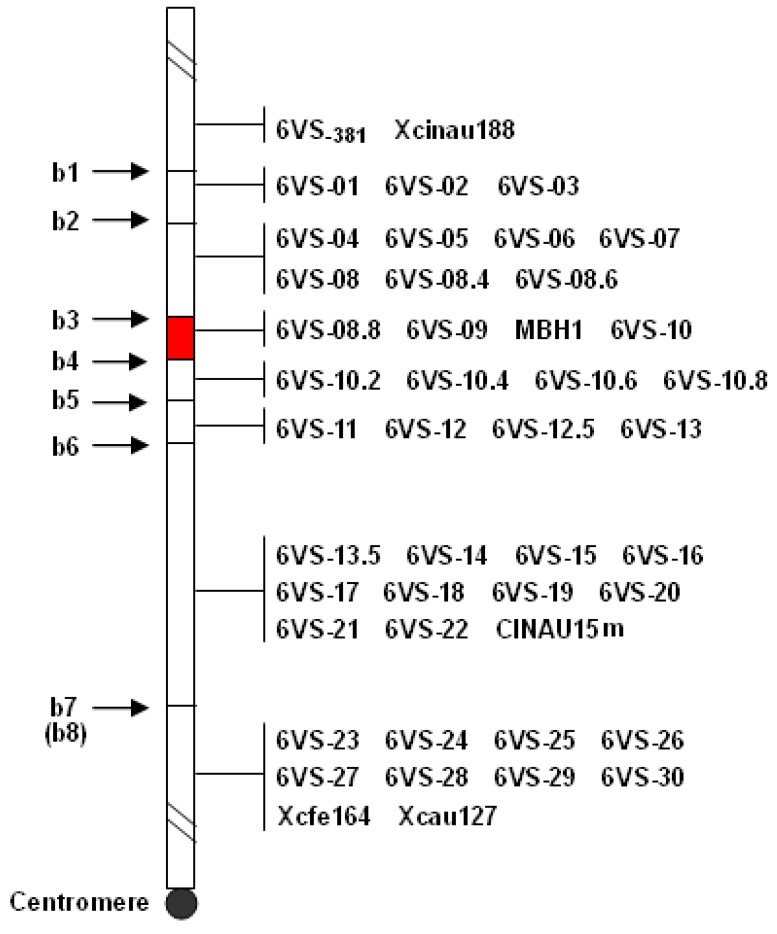
A physical map of 6VS. The breakpoints are shown in the left, and the 6VS-specific DNA markers are shown in the right. The order of the markers is according to the genomic locations of their corresponding orthologous genes in *Brachypodium*. The red box carries *Pm21*.

The amplification patterns of the representative markers were shown in Figure 1. The results revealed that there are 10 breakpoints on 6VS, designated as b1–b10 according to the direction from the telomere to the centromere (Figure 2, Table 1). Among them, b1 and b10 were in NAU419, b4 and b8 were in NAU418, and b3 and b5 were in Y18-S6. The breakpoints in del.6VS-1 (FL0.58) and del.6VS-1 (FL0.45) were named herein as b2 and b9, respectively. The result showed that b2 and b3 were flanked by the markers 6VS-03 and 6VS-04, and b9 and b10 were flanked by CINAU15m and 6VS-23. Therefore, b2 and b3, and b9 and b10 were close to each other, respectively. 

**Figure 3:**
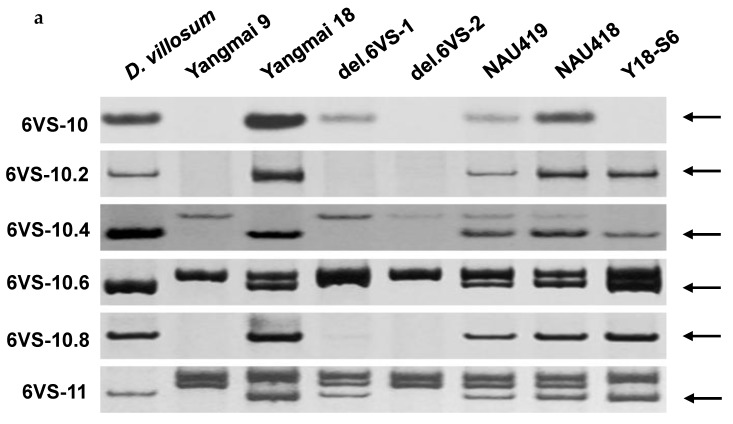

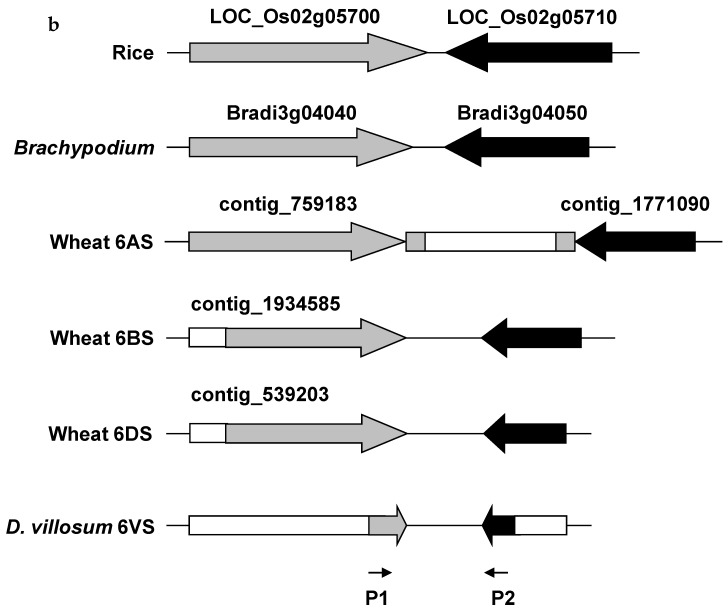
A newfound deletion region of 6VS in del.6VS-1. (**a**) Amplification patterns of the markers 6VS-10, 6VS-10.2, 6VS-10.4, 6VS-10.6, 6VS-10.8, and 6VS-11. The polymorphic DNA bands are pointed by arrows. All the markers 6VS-10.2, 6VS-10.4, 6VS-10.6 and 6VS-10.8 in the bin FLb5–b6, flanked by 6VS-10 and 6VS-11, were deleted in del.6VS-1; (**b**) Linkage relationship between the markers 6VS-10.8 and 6VS-11 in different (sub) genomes. The corresponding orthologous genes of 6VS-10.8 and 6VS-11 are indicated by grey and black arrows, respectively. The unknown sequences are shown in white boxes. On wheat 6AS, the grey boxes at the ends of contig_759183 and contig_1771090 indicate retrotransposon-like repeat sequences of wheat genome. On *D. villosum* 6VS, the linkage relationship of the corresponding genes of 6VS-10.8 and 6VS-11 were detected by PCR using the primers P1 and P2, and then confirmed by sequencing.

Unexpectedly, all the polymorphic bands of the markers 6VS-10.2, VS-10.4, 6VS-10.6, and 6VS-10.8 were absent in del.6VS-1 (Figure 3a), which implied that there was an additional deletion in del.6VS-1. Comparative analysis showed that the corresponding genes of the markers 6VS-10.8 and 6VS-11 were all linked on the short arms of chromosome 2 (2OsS) in rice and chromosome 3 (3BdS) in *Brachypodium*, and on wheat 6BS and 6DS, except a disruption caused by retrotransposon-like repeat sequence on wheat 6AS. To detect the linkage relationship of the two genes in *D. villosum*, a pair of primers, P1/P2, was designed according to the conserved sequences between wheat and *Brachypodium*, and used to amplify the potential fragment. Sequencing analysis showed that the ends of PCR products matched with the corresponding genes of 6VS-10.8 and 6VS-11, respectively (Figure 3b), demonstrating that the corresponding genes of the two markers are also physically linked in *D. villosum*. Taken together, a new interstitial deletion on 6VS was confirmed in del.6VS-1, and the corresponding breakpoints were then designated as b6 (close to b5) and b7. 

Therefore, chromosome 6VS could be divided into eight physical bins, viz., FL0–b9/b10, FLb8–b9/b10, FLb7–b8, FLb5/b6–b7, FLb4–b5/b6, FLb2/b3–b4, FLb1–b2/b3, and FLb1–1.00 (Figure 2). Because both NAU418 carrying the region FLb4–b8 and del.6VS-1 lacking the bin FLb5/b6-b7 are still immune to powdery mildew, *Pm21* can be mapped to the small bin FLb4–b5/b6 or FLb7–b8. Given that the deletion line Y18-S6 lacking of the bin FLb3–b5 is susceptible to powdery mildew, it was concluded that *Pm21* is located on the bin FLb4–b5/b6.

**Figure 4:**
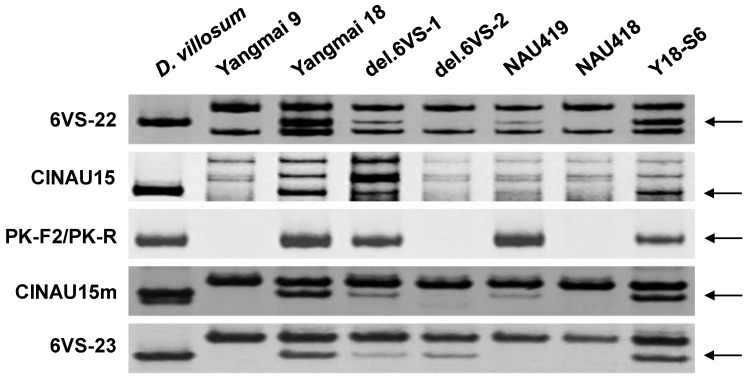
PCR amplification patterns of the markers 6VS-22, CINAU15, PK-F2/PK-R, CINAU15m, and 6VS-23. The polymorphic DNA bands are indicated by arrows.

*Stpk-V*, encoding a serine/threonine protein kinase, was previously reported to be a key member of the *Pm21* locus [2]. However, this study showed that the dominant marker PK-F2/PK-R [12] and the co-dominant markers CINAU15 [13] and CINAU15m, each of them derived from *Stpk-V*, together with the flanking markers 6VS-22 and 6VS-23, were all assigned to FLb8–b9/b10, rather than FLb4–b8 which appears in NAU418 (Figure 2 and Figure 4). 

### 2.2. Comparative Genomics Analysis of 6VS FLb4–b5/b6

The physical bin FLb4–b5/b6 carrying *Pm21* was flanked by the markers 6VS-08.6 and 6VS-10.2. The orthologs of the corresponding genes of 6VS-08.6 and 6VS-10.2 were Bradi3g03850 (2,587,675–2,590,972) and Bradi3g04010 (2,711,658–2,714,402) in *Brachypodium*, respectively. Hence, the orthologous region of 6VS FLb4–b5/b6 was narrowed to a 117.7 kb genomic region harboring 19 genes in *Brachypodium*. In rice, due to having no ortholog of 6VS-08.6 in the target region, the region between the corresponding genes LOC_Os02g05610 (2,728,848–2,734,454) and LOC_Os02g05670 (2,774,720–2,772,143) of the markers 6VS-08.4 and 6VS-10.2 was considered as the orthologous region, which is 37.7 kb in length harboring five genes. 

In the orthologous regions of 6VS FLb4–b5/b6, *Brachypodium* and rice had four conserved genes, which encode an exocyst complex component EXO70A1, a serine/threonine protein phosphatase 2C (PP2C), a homeobox-leucine zipper protein HOX26, and an DEAD (Asp-Glu-Ala-Asp)-box ATP-dependent RNA helicase (DARH), respectively (Table 2). Among them, except *DARH*, the other three genes—*EXO70A1*, *PP2C*, and *HOX26*—have their orthologs on the short arms of wheat homoeologous group 6 and have been used to develop the markers 6VS-08.8, 6VS-09, and 6VS-10, respectively (Figure 2). 

Recently, a conserved resistance gene analog (RGA) locus between wheat and *Brachypodium* was confirmed to co-segregate with *Pm21* in a genetic mapping population derived from a cross between resistant and susceptible *D. villosum* lines [7]. In this study, comparative analysis showed that the above RGA locus was still in wheat and *Brachypodium* orthologous regions of FLb4–b5/b6. Molecular detection using the markers MBH1 and 6VS-09.4 [3,7], both derived from this RGA locus, indicated that it could also be assigned to the bin FLb4–b5/b6 (Figure 1 and Figure 2).

## 3. Discussion

*Pm21*, one of most effective powdery mildew resistance genes, is highly resistant to all known *Bgt* isolations. However, due to the lack of recombination between the alien chromosome 6VS and wheat homoeologous chromosomes, it is infeasible to genetically map *Pm21* in wheat background. In the past, several genetic stocks involving in chromosomal structural changes of 6VS have been reported [4,6,7,11], which allows to physically map *Pm21* in common wheat. In the present study, the structural variations in these genetic stocks were scanned by 45 6VS-specific markers, and a total of 10 chromosomal breakpoints were found. Then, chromosome 6VS was divided into 8 distinguishable physical bins, and *Pm21* was finally physically mapped to the bin FLb4–b5/b6, flanked by the markers 6VS-08.6 and 6VS-10.2. In our recent work, the genetic interval carrying *Pm21* was confirmed to be flanked by the markers 6VS-08.4b and 6VS-10b [7]. Consequently, the physical location of *Pm21* is approximately consistent with the genetic interval. 

NAU418 and NAU419 are two small alien translocation lines resistant to powdery mildew [6]. Here, the results indicated that NAU418 carries the bin FLb4–b8, the size of which corresponds to 224.7 kb (Bradi3g03860-Bradi3g04140) in *Brachypodium* and 142.1 kb (LOC_Os02g05620-LOC_Os02g05840) in rice, respectively, while NAU419 contains the bin FLb1–b9/b10, almost covering the bin FL0.45–0.58 defined previously [2,4]. Hence, NAU418 carries smaller alien chromosomal segment than NAU419, suggesting that NAU418 might have more important value for wheat breeding. Chen et al. reported that the marker CINAU15 derived from *Stpk-V*, which was confirmed to be a key member of the *Pm21* locus since whose overexpression provided high resistance to powdery mildew in transgenic wheat, also appeared in NAU418 [6]. However, in this study, it was found that *Stpk-V* is not located in NAU418 by molecular analysis. The confusion may be caused by low resolution of the corresponding marker CINAU15, which is subject to false positive detection. 

In the past few decades, several powdery mildew resistance genes—such as *Pm12*, *Pm13*, and *Pm20* [14,15,16]—have been transferred from wild relatives into common wheat; However, like 6VS carrying *Pm21*, the corresponding translocated chromosomal arms, 6SS, 3S^l^S, and 6RL, cannot recombine with the ones of wheat. Hence, till now, these genes have not been fine mapped by using classic genetic mapping strategy in wheat background, which hampers the cloning and utilization of these important genes. This study demonstrated that physical mapping using genetic stocks involving in chromosomal structural variations could be an alternative and efficient method for locating these alien genes.

## 4. Materials and Methods 

### 4.1. Plant Materials

The common wheat varieties Yangmai 9 and Yangmai 18 were developed in Yangzhou Academy of Agricultural Sciences (YAAS). Yangmai 9 is highly susceptible to powdery mildew. Yangmai 18 carrying a pair of T6AL.6VS is immune to powdery mildew. The *D. villosum* accession and its derived resistant translocation lines NAU418 and NAU419 [6], resistant deletion lines del.6VS-1 (FL0.58) [11] and susceptible del.6VS-2 (FL0.45) [4] were kindly provided by Professor Peidu Chen, the Cytogenetics Institute, Nanjing Agricultural University. The susceptible deletion line Y18-S6 was obtained from Yangmai 18 mutagenized with EMS in our group [7].

### 4.2. Development of 6VS-Specific Markers

6VS-specific markers were published in previous works or newly developed here using the CISP (conserved-intron scanning primers) and CISP-IS (CISP combined with intron sequencing) strategies based on the collinearity relationship between *Brachypodium*, rice, and Triticeae species [5,17]. The newly developed markers were listed in Table 3.

### 4.3. DNA Extraction and PCR

Genomic DNA was isolated from fresh leaves of the seedlings by the CTAB (cetyl trimethyl ammonium bromide) method [18]. PCR amplification was performed in Peltier thermal cycler (Bio-Rad, Hercules, CA, USA) in 25 μL volume containing 1× PCR buffer, 0.2 mM of each dNTP, 2 μM of each primer, 1 Unit of *Taq* DNA polymerase, and 50 ng genomic DNA. PCR was carried out with an initial denaturation at 94 °C for 3 min, 35 cycles of 20 s at 94 °C, 30 s at 60 °C, 1 min at 72 °C, and a final extension for 5 min at 72 °C. PCR products were separated in 6% or 8% non-denaturing polyacrylamide gels, silver stained, and photographed.

### 4.4. Physical Mapping of Pm21

Using NAU418, NAU419, del.6VS-1, del.6VS-2, and Y18-S6 involving in structural variation of 6VS as materials, polymorphic DNA markers were assigned to different 6VS bins, and then a high-density physical map was obtained. 

### 4.5. Comparative Genomics Analysis of the Pm21 Locus

The genome sequences of *Brachypodium*, rice and wheat were obtained from the *Brachypodium distachyon* genome assemblies v2.0 (http://www.brachypodium.org), the rice genome pseudomolecule release 7 (http://rice.plantbiology.msu.edu) and the IWGSC (The International Wheat Genome Sequencing Consortium) Sequence Repository (http://wheat-urgi. versailles.inra.fr), respectively. Genes were predicted by using the FGENESH tool (http://linux1.softberry.com), and then re-annotated by using the BLAST program (http://blast.ncbi.nlm.nih.gov/Blast.cgi) and the SMART program (http://smart.embl-heidelberg.de). 

## Figures and Tables

**Table 1:** Chromosomal breakpoints (b1–b10) in different genetic stocks.

Genetic Stock	Treatment	Chromosomal Breakpoint	Flanking Markers
del.6VS-1	Irradiation ^a^	b2 (FL0.58)	6VS-03/6VS-04
		b6	6VS-10/6VS-10.2
		b7	6VS-10.8/6VS-11
del.6VS-2	Irradiation	b9 (FL0.45)	CINAU15m/6VS-23
NAU418	Irradiation	b4	6VS-08.6/6VS-08.8
		b8	6VS-13/6VS-13.5
NAU419	Irradiation	b1	Xcinau188/6VS-01
		b10 (Close to b9)	CINAU15m/6VS-23
Y18-S6	EMS	b3 (Close to b2)	6VS-03/6VS-04
		b5 (Close to b6)	6VS-10/6VS-10.2

^a^ As described, the deletion line del.6VS-1 was spontaneously formed from the wheat-*D. villosum* addition line DA6V#2 [11]; however, in fact, it may be obtained after treatment with ^60^Co γ-rays (Prof. Peidu Chen, personal communication).

**Table 2:** Gene annotation in *Brachypodium*, rice and wheat orthologous regions of the bin FLb4–b5/b6 carrying *Pm21.*

Marker	*Brachypodium*	Rice	Wheat	Gene Annotation
6VS-08.6	Bradi3g03850		6AS_contigs_4399884 6BS_contigs_2953789 6DS_contigs_2117578	Eukaryotic translation initiation factor
6VS-08.8	Bradi3g03860	LOC_Os02g05620	6AS_contigs_4431592 6BS_contigs_2953283 6DS_contigs_2092656	Exocyst complex subunit EXO70-like protein
6VS-09	Bradi3g03870	LOC_Os02g05630	6AS_contigs_4363243 6BS_contigs_2962596 6DS_contigs_2093935	Serine/threonine protein phosphatase 2C
MBH1	Bradi3g03874 ^a^		Six contigs ^a^	Disease resistance protein
6VS-09.4	Bradi3g03878 ^a^		Six contigs ^a^	Disease resistance protein
	Bradi3g03882 ^a^		Six contigs ^a^	Disease resistance protein
	Bradi3g03886			Unknown protein
	Bradi3g03890			Unknown protein
	Bradi3g03900			Unknown protein
	Bradi3g03910			Cytochrome P450
	Bradi3g03920			Unknown protein
	Bradi3g03930			Poly(A) polymerase
	Bradi3g03935 ^a^		Six contigs ^a^	Disease resistance protein
	Bradi3g03940		6AS_contigs_4428294 6BS_contigs_2926507 6DS_contigs_2114667	Photosystem II protein J
	Bradi3g03945		6AS_contigs_4428294 6BS_contigs_2926507 6DS_contigs_2114667	Photosystem II cytochrome b559 alpha subunit
	Bradi3g03950			Unknown protein
	Bradi3g03957		6AS_contigs_4431958 6BS_contigs_2953283 6DS_contigs_2081863	Polyubiquitin
6VS-10	Bradi3g03970	LOC_Os02g05640	6AS_contigs_4429249 ^b^ 6BS_contigs_1424756 ^b^	Homeobox-leucine zipper protein HOX26
			6DS_contigs_2087435 ^b^	
		LOC_Os02g05650		Unknown protein
	Bradi3g03980		6AS_contigs_2062052	Unknown protein
			6DS_contigs_2110435	
	Bradi3g03990	LOC_Os02g05660		DEAD-box ATP-dependent RNA helicase
6VS-10.2	Bradi3g04000	LOC_Os02g05661	6AS_contigs_4428740 6DS_contigs_2082952	Unknown protein

^a^ Four RGAs (Bradi3g03874, Bradi3g03878, Bradi3g03882 and Bradi3g03935) in *Brachypodium* are highly homologous with six contigs (6AS_contigs_4363243, 6AS_contigs_4428238, 6BS_contigs_3017519, 6BS_contigs_2958212, 6DS_contigs_2091261 and 6DS_contigs_1402211) on the short arm of wheat homologous group 6; ^b^ Multiple contigs were matched on the short arms of wheat homologous group 6.

**Table 3:** 6VS-specific markers newly developed in this study.

Marker	Strategy	Primer Sequence (5′ → 3′)	Annealing Temperature (°C)	Size of Polymorphic Band (bp)
6VS-08.6	CISP-IS	F: GATCTCGTTTTAGTCCTGAGCCTGT	60	604
		R: CCGAGTGAAGTCTGCTCATCCAGT		
6VS-12.5	CISP	F: CCGGCGACGCGCACTAC	60	391
		R: GTACTTGTCCGCGAACTCGAAC		
6VS-13.5	CISP	F: TGCCTCATCACCCCAGTGAA	60	287
		R: TGCAGTAGGTGGCATAGAGCAAG		

F: forward primer; R: reverse primer.
